# Allergenic protein-induced type I hypersensitivity models: a review

**DOI:** 10.3389/falgy.2024.1481011

**Published:** 2024-10-17

**Authors:** Yanhua Feng, Liangyu Xu, Jinming Zhang, Jinlian Bin, Xialing Pang, Sheng He, Lei Fang

**Affiliations:** ^1^ Paediatric Department, Maternal and Child Health Hospital of Guangxi Zhuang Autonomous Region, Guangxi Clinical Research Center for Pediatric Diseases, Nanning, China; ^2^ Institute of Translational Medicine, Medical College, Yangzhou University, Yangzhou, China; ^3^ Guangxi Key Laboratory of Reproductive Health and Birth Defects Prevention, Maternal and Child Health Hospital of Guangxi Zhuang Autonomous Region, Nanning, China; ^4^ Jiangsu Key Laboratory of Experimental & Translational Non-Coding RNA Research, Yangzhou University Medical College, Yangzhou, China

**Keywords:** type I hypersensitivity, allergy, IgE, ovalbumin, RBL-2H3

## Abstract

**Context:**

Type I hypersensitivity affects approximately one-third of the global population. As the pathophysiology underlying the development of type I hypersensitivity (asthma, food allergy, and anaphylactic shock, etc.) is complex and heterogeneous, animal model studies continue to be the key to identifying novel molecular pathways and providing therapeutic strategies.

**Objective:**

Selection of the animal model should be done with careful consideration of the protocol variables, animal species, and strains to accurately reflect the clinical symptoms typical of humans.

**Methods:**

The following databases were searched: PubMed and Web of Science.

**Results and conclusion:**

Foreign allergens include allergenic proteins and chemical haptens. This review summarizes the various methods used for designing animal models of common allergenic protein-induced type I hypersensitivity, namely, passive anaphylaxis model, active systemic anaphylaxis/anaphylaxis shock model, food allergy model, asthma model, and IgE-mediated cell models. Additionally, we summarize shrimp tropomyosin-induced type I hypersensitivity models from our previous studies and discuss their advantages and limitations compared with that of ovalbumin-induced models.

## 1 Introduction

Hypersensitivity reactions—often referred to as allergies or allergic reactions—constitute over-reactions of the immune system. The four primary classification of hypersensitivity reactions are types I, II, III, and IV; despite some modifications and extensions, this classification is still used today (1). The type I hypersensitivity reactions are the most commonly occurring ones, and they manifest as physiological dysfunction or tissue or cell damage upon subsequent exposure to the same allergen after an initial response. These reactions are driven by allergen-specific IgE antibodies that activate mast cells to produce specific effects (2). Type II and III hypersensitivity reactions are mediated by IgG or IgM antibodies, which activate the complement system. Type II is a cytotoxic reaction in which antibodies predominantly bind to cell-surface antigens on their own cells, followed by phagocytosis or destruction. In type III reactions, the antibodies bind to soluble antigens and form immune complexes through crosslinking, which results in tissue damage. Type IV reactions are delayed reactions mediated by T lymphocytes, where macrophages are activated by Th1 cells, followed by Th2-associated eosinophilic inflammation and direct damage to tissues by cytotoxic T cells (3).

Type I hypersensitivity leads to atopic and allergic diseases, which are immediate reactions to foreign allergen challenge. A wide range of allergens exist. For example, respiratory allergens include dust mites, fungal elements, pollen, and cockroach extracts; they are pivotal for the development of both allergic rhinitis and asthma (4). Dietary allergenic antigens include milk, peanuts, and shellfish, leading to protein-induced enterocolitis syndrome (5). Insect venoms contain a wide range of allergens (6). IgE-mediated drug hypersensitivity is most commonly induced by precipitating drugs such as antibiotics (particularly beta-lactams). In other cases drug may act as haptens that form covalent hapten–carrier links and initiate during sensitization (7). Generally, anaphylactic shock is triggered by drug, food, or venom and represents the most severe systemic hypersensitivity reaction (8).

Type I hypersensitivity is characterized by an IgE-mediated response. Generally, IgE is a protective antibody, particularly in the immune response against parasitic infections. Upon initial exposure to an allergen, native CD4^+^ T cells are converted into Th2 cells, which secrete IL-4 and IL-13. The engagement of CD40 on B cells with the surface CD40 ligand expressed on CD4^+^ T cells triggers IL-4-driven IgE isotype switching (9). IgE binds to specific receptors (Fc*ε*RI) on mast cells and basophils. Upon re-exposure to the same allergen, Fc*ε*RI on mast cells and basophils cross-links with the IgE and antigen, which initiates cascades leading to cell degranulation, synthesis and secretion of lipid mediators and cytokines (IL-4 and IL-13), and CD40 ligand expression, which further amplifies IgE-mediated responses (10).

Thus, Type I hypersensitivity is induced by allergenic proteins or chemical haptens. Various types of *in vivo* and *in vitro* models have been used in laboratory experiments to explore the molecular mechanism underlying type I hypersensitivity and test novel therapeutic approaches. This review summarizes various methods used to establish animal models of common allergenic protein-induced type I hypersensitivity and IgE-mediated cell models and discusses their advantages and limitations. However, hapten-induced dermatitis, asthma, and drug anaphylaxis models have not been discussed in this review.

## 2 Animal models of type I hypersensitivity

### 2.1 Animals

Animal models of allergic reactions are commonly established in guinea pigs, rats, and mice. Guinea pigs have been used for asthma research for several decades owing to the high anatomical and physiological similarities between the airways of humans and guinea pigs. The important similarity is that the acute bronchoconstriction response to allergens is largely mediated by histamine and leukotrienes. Guinea pigs are recommended for preclinical research on allergen-induced airway hyper-responsiveness (11). However, allergen-induced IgG1 is associated with a high degree of early airway smooth muscle contraction in the guinea pig, leading to a higher level of histamine release by the mast cells (12). Thus, guinea pig models are suited for investigating mast cell-dependent reactions. Among rat strains, Sprague–Dawley (SD) rats are typically used for studying passive cutaneous anaphylaxis (PCA) reactions (13). Brown Norway (BN) rats are used for exploring the allergenicity of food proteins (14) and asthma studies because of their high sensitivity (15). Mice have also been used as allergy models, and different species or strains show variability in terms of physiology and immunology. BALB/c mice are the most commonly used mouse strain in antigen challenge models because they develop a Th2-biased immunological response (16). C57BL/6 and A/J mice have been successfully used as experimental models for allergic asthma (17). C57BL/6 mice are commonly used to establish transgenic or knockout models that provide information about specific genes involved in human diseases. In the OVA-induced asthma model, C57BL/6 mice display a higher eosinophil percentage in the bronchoalveolar lavage fluid (BALF) than that of BALB/c mice, whereas BALB/c mice show an increased lung mast cell count and airway hyperresponsiveness (AHR) than that observed in C57BL/6 mice (18). A/J mice exhibit higher levels of AHR and reactivity to methacholine than do BALB/C and C57BL/6 mice (19).

Humanized mice, that is, mice reconstituted with human immune cells, are a novel tool for studying the immune response during type I hypersensitivity. Eschborn M developed a humanized mouse model of allergen-induced IgE-dependent gut inflammation in PBMC-engrafted immunodeficient mice (20). Another humanized mouse model of immunodeficient *γ*c-deficient mice expressing transgenes for human stem cell factor, granulocyte-macrophage colony-stimulating factor, and IL-3 developed mature functional human mast cells. Human mast cells in mice were sensitized with patient-derived IgE monoclonal antibodies specific for peanut (*Arachis hypogaea*) allergen 2, which induced fatal anaphylaxis on exposure to the peanut allergen (21). These humanized mice are useful for recapitulating human IgE-mediated or mast cell-induced anaphylaxis.

### 2.2 Allergens

The classical antigens used for Type I allergic reaction models include ovalbumin (OVA) and 2,4-dinitrophenol (DNP). DNP is a low-molecular-weight chemical hapten that is conjugated to carrier proteins such as human serum albumin or bovine serum albumin. Anti-DNP-IgE and anti-DNP-human serum albumin (or anti-DNP-bovine serum albumin) antibodies have been used in PCA or mast cell models (22). Nevertheless, OVA is most commonly used, primarily for practical reasons such as low costs, high purity, and commercial availability of antibodies (OVA-specific IgE/IgG) and transgenic mice (OVA-specific transgenic mice). OVA is an allergenic protein that is commonly used to induce allergic bronchospasm in guinea pigs and PCA, asthma, and food allergies in rodent models. However, immunization with OVA in mice does not always induce high levels of IgE and may require re-exposure to allergens. In fact, OVA-induced allergy requires the use of both OVA and Th2-adjuvant aluminum hydroxide to sensitize the mice (23).

Antigens that cause food allergies are typically protein antigens such as milk, eggs, wheat, fish, shellfish, peanuts, walnuts, and soybeans (24). To better mimic the gastrointestinal lesions observed in human food allergies, animals are often sensitized via the oral route. Due to their oral tolerance, cholera toxin and staphylococcal enterotoxin B (SEB) are generally used as mucosal adjuvants to enhance the induction of antigen-specific IgE. Subcutaneous sensitization has been proposed as an alternative approach for inducing gastrointestinal symptoms of food allergies in animals (25).

The allergen inhalation challenge is a useful clinical model for evaluating allergic airway diseases that are triggered by the inhalation of food allergens or airborne allergens (26). Although OVA is widely used in asthma experiments, it is not a relevant aeroallergen for human asthma. The clinical relevance of aeroallergens such as house dust mites (HDM) has been investigated. Additionally, HDM sensitize animals via the airways and do not require adjuvants (27) similar to that of the human sensitization route.

### 2.3 Passive anaphylaxis model

Passive anaphylactic models are broadly categorized into local cutaneous and systemic reactions. The principle of PCA is the same as that of anti-allergen IgE sensitization and allergen re-exposure in mast cells. The model is generated by administering a subcutaneous injection of mouse IgE-rich antiserum or anti-allergen-IgE monoclonal antibodies (28) at the back or dorsal portion of either ears in rats (29) or paw of mice (30). After 24–48 h, Evans blue dye containing the antigen is administered via the tail vein. As the local vascular permeability increases owing to the allergic reaction, the degree of the reaction is assessed by measuring the diameter of the local blue skin spots or observing the extent of blue skin staining. However, this parameter is non-quantitative and may not reflect a dose-response relationship for evaluating drug efficacy; hence, some researchers have collected local skin samples and quantitatively determined Evans blue absorbance using formamide (22) or acetone–saline extraction (28). Allergen-dependent paw swelling is confirmed by measuring changes in hind paw width using digital calipers (31).

In the passive systemic anaphylaxis (PSA) model, sensitized animals are injected with anti-allergen IgE monoclonal antibodies at the tail vein, followed by administration of the antigen via the tail vein after 24 h, which causes a decline in body temperature and an increase in symptom scores with respect to antigen-specific IgE-dependent PSA reactions (32). In our previous study, mice were intravenously sensitized with anti-shrimp tropomyosin monoclonal IgE. After 24 h, the mice were challenged intravenously with shrimp tropomyosin. After 30 min, rectal temperature decreased by 1.6℃ (33). The degree of PSA was determined by measuring histamine, leukotrienes, and prostaglandins levels in the blood, which were collected through cardiac puncture 5 min after allergen challenge (34).

### 2.4 Active systemic anaphylaxis (ASA)/anaphylaxis shock model

The ASA is commonly used to evaluate drug safety in guinea pigs. The drug is administered via multi-point injection into the muscles three times every alternate day. On days 14 and 21, an intravenous drug challenge is administered to evaluate the degree of systemic reactions (35). Additionally, ASA is elicited by injecting a protein antigen into the tail vein of mice immunized with the antigen and adjuvant for to 2–4 weeks. Similar systemic symptoms, including shock, develops during ASA therapy. Antigens and adjuvants induce IgG1/2 and IgE in ASA mice. IgG-class antibodies recognize and bind to Fc gamma receptors (Fc*γ*Rs) that are expressed on mast cells, neutrophils, monocytes and macrophages in humans (36). In mouse models, antigens induce basophils (37), macrophages, and neutrophils (38) to release PAF by activating Fc*γ*Rs, accounting for IgG-induced anaphylaxis. A higher mortality rate is observed during ASA testing than during PSA testing. Moreover, clinical surrogate measurements such as rectal temperature measurements and behavior scales were used similar to that used for PCA. The central temperature variation (ΔT) observed during the 30 min following the challenge was classified according to the standard described by Jonsson F et al. (39) as Grade 1: no shock, 1°C > ΔT ≥ −1°C, no mortality; Grade 2: mild shock, −1°C > ΔT ≥ −4°C, no mortality; Grade 3: severe shock, −4°C > ΔT, and/or mortality.

### 2.5 Food allergy models

Skin prick tests and serum allergen-specific IgE levels are routinely used for clinical diagnosis of food allergies. Alternatively, basophil activation test is performed, where flow cytometry is used to measure the expression of both CD63 and CD203c on the surface of basophils following stimulation with food allergens (40). In animal models, the sensitization routes may be intragastric, epicutaneous, subcutaneous, intraperitoneal, or inhalational. However, oral sensitization is the primary route in food allergy models as it is similar to the natural oral route in humans. As an adjuvant, cholera toxin disrupts the intestinal barrier and promotes mucosal immune reactions. The combination of whey protein (15–20 mg) or β-lactoglobulin (1 mg) (to represent cow milk allergen) and 10 μg of cholera toxin is often administered for 5 weeks for sensitization to generate a mouse model of cow milk allergy. Notably, a whey-free diet was provided to the mice for at least 1 week prior to initiating the sensitization procedure (41, 42). Shrimp could trigger severe food allergies with tropomyosin as the major cross-reactive allergen to other shellfish. The quantity of shrimp tropomyosin required for sensitization varies from 30 to 200 μg (43, 44). Peanut is another common food allergen. In addition to using peanut allergen with cholera toxin for sensitization, Joseph J. Dolence developed a mouse model for inhalation-based peanut allergen sensitization. A large number of follicular helper T cells have been detected in the mediastinal lymph nodes of allergen-sensitized mice, and the IL-1 pathway is involved in the Tfh response to peanut allergen (45). Although OVA-specific IgE levels were elevated in mice that were orally immunized with OVA–cholera toxin combination, high serum IL-4 levels, increased mast cell counts in the jejunum, and anaphylaxis were not detected following oral challenge. Upon epicutaneous sensitization with OVA, mice exhibited IgE-mediated mast cell expansion, intestinal allergy, and anaphylaxis, as evidenced by decreased core temperature and increased serum mMCP-1 levels (46). Keiko Kameda developed a food allergy mouse model using 1 mg of chicken egg ovomucoid or cow milk casein in epicutaneous sensitization process without adjuvants (47), indicating that the skin may be an efficient route of sensitization to food antigens. Thus, the protocol design for a mouse food allergy model including the allergen, adjuvant, route, dose, timing of sensitization and challenge exposures, endpoint selected, and outcome of the study is described in Table 1.

**Table 1:** Protocol design of mouse food allergy model.

Mice	Sensization	Challenge	Response
BALB/c mice	Whey protein (+10 μg cholera toxin), i.g., 15 mg, days 7, 14, 21, 28, 35	β-lactoglobulin, i.g., 60 mg, days 42, 64	Mouse mast cell protease-1 (mMCP1) and β-lactoglobulin -specific IgE, IgG1, and IgG2a in plasma ↑ (41)
C3H/HeJ mice	Whey protein (+10 μg cholera toxin), i.g., 20 mg, days 7, 14, 21, 28, 35	Intradermally and orally challenged with 10 µg or 20 mg whey respectively, day 40	*Δ*Ear pinnae thickness ↑ Serum whey-specific IgG1 and BLG-specific IgG1 ↑ IL-10 in ex vivo re-stimulation of splenocyte ↑ (48)
C57/BL6J mice	β-lactoglobulin (+10 μg cholera toxin), i.g., 1 mg, once a week for 5 weeks	OVA, i.g., 50 mg, day 36	Anaphylactic symptom score ↑ Serum IgE and IgG1 ↑ (42)
BALB/c mice	shrimp protein (+aluminum hydroxide), s.c., 100 µg, days 0, 14	Water containing shrimp proteins (5 mg/ml), from day 21–35	Serum total IgE, anti-shrimp IgE, IgG1 and IgA ↑ IL-5 and IL-10 production mesenteric lymph node cell ↑ Eosinophils in intestinal mucosa ↑ (49)
BALB/c mice	Shrimp tropomyosin (+aluminum hydroxide), i.p., 50 μg, days 1, 7, 14	Shrimp tropomyosin, i.p., 100 μg, Days 22, 25, 31, 34, 37, 40, 43	IL-4, IL-5, IL-13 released from splenocytes ↑ IL-10 and IFN-γ levels released from splenocytes ↓ Villous atrophy, destruction of the mucosal lining and infiltration of inflammatory cells in duodenum (50)
BALB/c mice	Shrimp tropomyosin (+10 μg cholera toxin), i.g., 200 μg, days 0, 7, 14, 21	Shrimp tropomyosin, i.g., 800 μg, Day 28	Anaphylactic shock score ↑ Specific IgE, IgG1, IgG2a in sera↑ Histamine and mMCP-1 in serum ↑ IL-4, IL-5, IL-13, IL-17A, IL-21, IL-22 in splenocyte ↑ IL-4 and IL-13 in the jejunum ↑ Goblet cell count in the jejunum, eosinophil and lymphocyte count in the duodenum and ileum ↑ (43)
C3H/HeJ	Peanut protein extract (+15 μg cholera toxin), i.g., 6 mg, days 0, 1, 2, 7, 14, 21, 28	Peanut protein extract, intradermal injection in both ear pinnae, 1 μg, day 64; 15 mg, i.g., day 70; 1 μg, i.p., day 77	*Δ*ear swelling ↑ Murine mast cell protease-1 in serum↑ Core body temperature ↓ Specific IgA, IgE, IgG1, and IgG2a levels in serum ↑ (51)
BALB/c mice	Peanut flour, 100 μg, i.n., twice/week for up to 4 week	Crude peanut extract, 2.5 mg, i.p., day 28	Rectal temperature ↓ Serum IgE, IgG1, IgG2a ↑ MCPT-1 levels in plasma ↑ IL-4eGFP ^+^ CD4^+^ T cells and ollicular helper T cells in mLNs ↑ (45)
BALB/c mice	OVA (+aluminum hydroxide), i.p., 50 μg, days 0, 14	OVA, i.g., 50 mg, days 28, 30, 32, 34, 36, 38, 40	Diarrhea OVA-specific IgE, histamine and mMCP-1 in serum ↑ IL-4 and IL-13 in the jejunum tissue ↑ (52)
BALB/c mice	Epicutaneously sensitization consisted of applying a 1 cm^2^ gauze containing 100 µg OVA, days 0–7, 21–28, 42–49	OVA, i.g., 100 mg, day 50	Body temperature ↓ Serum OVA-specific IgE, mMCP1 ↑ IL-4 and IL-13 secretion by splenocytes ↑ Total jejunal mast cell numbers ↑ (46)

### 2.6 Allergic asthma models

Human allergic asthma and rhinitis are triggered by aeroallergens and foodborne allergens. Most animal models of asthma and rhinitis are based on initial sensitization to antigens, followed by a local challenge. None of the currently used animal models emulate all the characteristics of allergic airway diseases. Guinea pigs are commonly used to induce immediate mast cell-dependent hypersensitivity reactions. Bronchial circulation, number of mast cells and mucus glands, and airway neural control in the lungs of guinea pig lungs are significantly similar to those described in humans. Notably, the immediate hypersensitivity reaction in the lung mimics that observed in humans and involves the activation of H1 and cysLT1 receptors (53). However, large doses of antigens induce severe immediate hypersensitivity and favor IgG production, whereas low doses favor mixed IgE and IgG production (54). Moreover, few protocols have been suggested for airway remodeling in guinea pigs. In rats, strains such as Wistar, SD, Fisher, and Lewis do not always develop an allergic response to IgE production, whereas Brown Norway (BN) rats have a high IgE response (55). Airway smooth muscle contraction elicited by allergens in rats is relatively weak and primarily mediated by serotonin, which differs between guinea pigs and humans (54).

BALB/c mice are commonly used to generate mouse models to evaluate Type 2 eosinophilic inflammation or airway remodeling. T2 asthma responds to steroid treatment. Briefly, 10–100 μg of OVA is used to elicit systemic sensitization through intraperitoneal administration for 2–3 weeks, primarily in combination with aluminum hydroxide. In the challenge phase, the animal is exposed to 1%–5% aerosolized OVA inhalation or OVA intranasal instillation (3–7 days for acute asthma or 4–6 weeks for airway remodeling). Large doses of OVA (500 μg) (56) or long-term aerosolized OVA inhalation challenge (57, 58) induce more severe subepithelial fibrosis, whereas low dose (5 μg) challenges favor bronchial epithelial thickening and mucus accumulation in the epithelium (56). Similarly, in our recent studies, shrimp tropomyosin was used as an alternative to OVA, and it elicited high IgE production, AHR, eosinophilic inflammation, Th2 response, basophil and M2 macrophage activation, and airway remodeling in mice (23, 59, 60). Complete Freund's adjuvant (CFA) favors IgG2a activation, indicating a Th1 type immune response (61). OVA/CFA-sensitized mice were developed to induce a non-T2 asthma phenotype, which is characterized by airway neutrophil-dominant inflammation and glucocorticoid insensitivity (62, 63). However, most foodborne allergens, including OVA and shrimp tropomyosin, do not induce airway inflammation or AHR in humans.

Worldwide, *Dermatophagoides pteronyssinus* (Der p) and *Dermatophagoides farina* (Der f) are the most common HDM allergens inhaled by humans. *Blomia tropicalis* is a mite species prevalent in both tropical and subtropical regions (64). Unlike OVA, HDM sensitization and challenge were achieved in mice without the use of adjuvants. Murine eosinophilic asthma is induced by intranasal exposure to *Blomia tropicalis* allergen without an adjuvant (65). Three different asthma phenotypes, namely, eosinophilic, mixed, and neutrophilic were induced in mice via different doses and routes of HDM (a mixture of Der p and Der f allergen extracts) sensitization and challenge. Mice were sensitized with three intraperitoneal injections of low-dose HDM extract and aluminum hydroxide and challenged with low-dose HDM for eosinophilic airway inflammation. To induce the mixed or neutrophilic phenotype, mice were intranasally sensitized and challenged with low or high doses of HDM, respectively (66). Wang et al. compared asthma induction in mice treated with Der p or Der f extracts. When compared with the Der f-challenged group, Der p-exposed mice exhibited higher BALF neutrophil counts and increased levels of IL-17A and MCPT-1 in lung homogenates, whereas the mice challenged with Der f exhibited a higher expression of MMP-9 and MMP-12 (67). Der p1 and Der f1 are the major allergen proteins in HDM. Recombinant purified Der p1 or Der f1 has been used to develop a capture IgE-ELISA to detect allergen-specific IgE responses in mite-allergic subjects (68). In addition, Der p1 or Der f1 challenged mice exhibited a similar phenotype of airway eosinophilic inflammation, as observed in mice induced with HDM. These recombinant proteins are often used to investigate the molecular mechanisms underlying allergen sensitization. CD163 is a Der p1-binding protein. Der p1 increases CCL24 secretion from bone marrow-derived macrophages in Cd163^−/−^ mice when compared to WT mice (69). Der p 2 drives airway Th2 inflammation via TLR4/Der p 2 interaction. Der p 2 and low-dose lipopolysaccharide (LPS) promote airway inflammation in WT mice, but not in TLR4^−/−^ mice (70).

**Figure 1:**
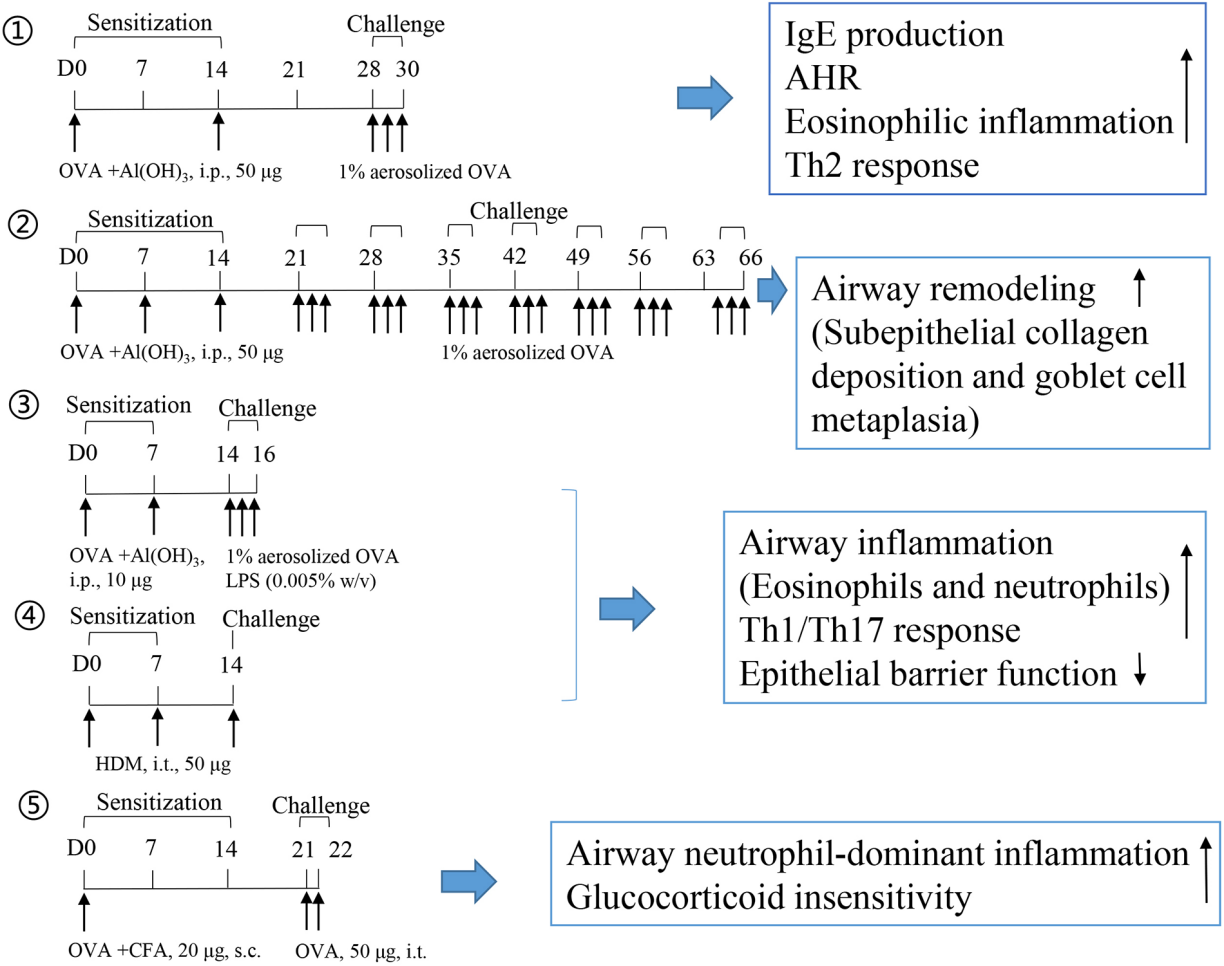
Experimental protocol of mouse asthma model.

LPS is a known risk factor for asthma exacerbation in humans. A combination of allergens and LPS was used to generate a severe asthma model. However, successfully establishing a severe asthma phenotype considerably depends on the allergen (OVA or HDM), dose of LPS (100 ng, 1 μg, or 10 μg), the starting time of LPS administration (in the sensitization or challenge phase), and LPS administration duration. A single dose of LPS (100 ng) in HDM-treated mice induces the accumulation of NET-releasing CXCR4^hi^ neutrophils in the lungs and promotes HDM-induced type 2 allergic airway inflammation (71). In OVA-induced asthma mice, LPS (1 μg) was intraperitonially injected before each OVA/alum sensitization. LPS treatment significantly alleviates Th2 allergic airway inflammation after OVA challenge (72). In our recent experiment, LPS (250 ng) was intranasally instilled to mice in the shrimp tropomyosin/alum sensitization phase, which reduced the eosinophil count in the BALF. A modified LPS/shrimp tropomyosin-induced severe asthma model was used in this study. Mice were intraperitonially injected with 20 μg of shrimp tropomyosin mixed with aluminum hydroxide on days 0, 7, 14, and 21. The combination of shrimp tropomyosin (20 μg) and LPS (1 μg) was intranasally instilled to mice on days 7, 14, and 21. In the challenge phase, the mice were intratracheally instilled with 50 μg of shrimp tropomyosin once on day 28 and exposed to an intranasal challenge with shrimp tropomyosin from days 29–31. Shrimp tropomyosin combined with LPS induced significant increase in eosinophil and neutrophil counts in the BALF, excessive mucus secretion, increase in IL-17A and IL-1β levels, and decrease in E-cadherin level, which are partially improved by dexamethasone treatment (unpublished data). Additionally, LPS has been used in the OVA challenge phase for determining the neutrophilic phenotype, which is characterized by increased neutrophil count in the BALF (>30% of the total cell count) (73, 74). However, LPS/allergen-induced peribronchial inflammation has often been complicated by the development of alveolitis (75), which differ from the airway lesions in asthmatic humans. Protocol design for mouse asthma model including allergen, adjuvant, route, dose, timing of sensitization and challenge exposures, endpoint selected, and outcomes of the study are described in Table 2. The experimental protocol, including OVA-induced eosinophilic asthma model (76) and airway remodeling model (57), OVA/LPS (73), or HDM-induced mix phenotype model (83), and OVA/CFA-induced neutrophilic asthma model (63), is shown in Figure 1.

**Table 2:** Protocol design of mouse asthma model.

Mice	Sensitization	Challenge	Response
BALB/c mice	OVA (+aluminum hydroxide), i.p., 50 μg, days 0, 14	1% aerosolized OVA, 20 min, days 28, 29, 30	Eosinophils, macrophages and lymphocytes in BALF ↑ IL-4, IL-5 and IL-13 in BALF ↑ (76)
BALB/c mice	OVA (+aluminum hydroxide), i.p., 50 μg, days 0, 14, 28	OVA, 5 or 500 μg inhalation, days 35, 36, 37, 40,	Eosinophils and neutrophils in the BALF↑ Bronchial epithelial thickening and mucus accumulation in the epithelium Or subepithelial fibrosis (56)
BALB/c mice	OVA (+aluminum hydroxide), i.p., 20 μg, days 1, 8	OVA, i.n., days 15, 16, 17	Lung and spleen weights ↑ Eosinophils in BALF ↑ Peribronchial inflammation and mucus production ↑ AHR ↑ IgE ↑ IL-4, IL-5 and IL-13 in BALF ↑ IL-25 and IL-33 in BALF and lung tissue ↑ (77)
BALB/c mice	OVA (+aluminum hydroxide), i.p., 20 μg, days 0, 14, 28	3% aerosolized OVA, 30 min, 3 straight days weekly from days 21 to 66	AHR ↑ IL-4, IL-5, IL-13 and OVA-specific IgE in BALF ↑ Neutrophils, macrophages, lymphocytes and eosinophils in BALF ↑ Subepithelial collagen deposition and goblet cell metaplasia (57)
C57BL/6 mice	OVA (+aluminum hydroxide), i.p., 10 μg, days 0, 12	1% aerosolized OVA, 30 min, days 25–31	AHR ↑ Serum IL-5, IL-13, and IL-33 ↑ Airways inflammation ↑ (78)
C57BL/6 mice	OVA (+aluminum hydroxide), i.p., 0.5 mg/ml, days 0, 12	OVA, i.n., 50 μg, once a day from days 18 to 23, every other day from days 25 to 47	AHR↑ Eosinophils and lymphocytes in BALF ↑ Airways inflammation and mucus production ↑ Subepithelial deposition of collagen, hypertrophy of airway smooth muscles, angiogenesis (79)
C57BL/6 mice	OVA (+aluminum hydroxide), 100 μg OVA s.c. and 100 μg OVA i.p., days 1, 8	5% aerosolized OVA 20 min, once a day from days 15 to 28 and every other day from days 30 to 42	AHR ↑ IL-4, IL-5 and IL-13 in BALF ↑ Eosinophils in BALF ↑ Mucus secretion and thickened bronchial smooth muscle ↑ ZO-1 and Occludin ↓ Claudin-2 ↑ (58)
C57BL/6 mice	Shrimp tropomyosin (+aluminum hydroxide), i.p., 20 μg, days 0, 7, 14	Shrimp tropomyosin, i.t., i.n., 40 μg, days 21–25	Lung weight/body weight ratio ↑ EPO↑ Eosinophilic inflammation and mucus hypersecretion ↑ IL4 and IL5 in the lung and spleen ↑ Lung eotaxin, Ccl-17, Muc5ac, Il-33, Arg-1, Ym-1, and Fizz-1 ↑ Arg-1+ cells in the lung and the BALF ↑ (60)
A/J mice	OVA (+aluminum hydroxide), 100 μg OVA i.p., days 1, 8, 15	1% aerosolized OVA, 30 min, days 22, 23, 24	AHR ↑ IgE ↑ Influx of inflammatory cells, goblet cells hyperplasia and mucin production ↑ Regulatory T cells and IL4-producing T cells ↑ (80)
C57BL/6 mice	OVA (+75 μl CFA), 20 μg, i.p., day 0	1% aerosolized OVA, days 21, 22,	Lung infiltrating inflammatory cells and mucus hypersecretion ↑ Neutrophils, eosinophils and lymphocytes in BALF ↑ IL-4, IL-5, IL-13, IL-17A, IL-22, IL-23 and IL-1β in the BALF ↑ (63)
C57BL/6 mice	OVA (+0.5 mg/ml CFA), 20 μg, s.c., day 0	OVA, 50 μg, by pharyngeal/laryngeal installation, days 21, 22	Neutrophils, eosinophils, plasmacytoid dendritic cells in the BALF ↑ Lung severe inflammation with bleeding IFNγ, OVA-specific serum IgE ↑ (62)
C57BL/6 mice	*B. tropicalis* extract, 100 μg, i.n., day 0	*B. tropicalis* extract, 50 μg, i.n., days 7, 9, 11, 14 and 16	Eosinophils in BALF ↑ AHR ↑ (65)
C57BL/6 mice	Der p1, 2 μg, i.p., days 1 and 8	Der p1, 10 μg, i.t., day 15	Eosinophils and CCL-24 level in BALF ↑ Mucous cell metaplasia↑ (69)
C57BL/6 mice	HDM, 1 μg, i.n., day 1	HDM, 10 μg, i.n., days 8, 9, 10 and 11	Airways inflammation ↑ CysLT, CCL17, and IL-6 production of bone marrow-derived macrophages ↑ (81)
C57BL/6 mice	HDM, 100 μg, i.n., day 0	HDM, 10 μg, i.n., days 7–11	Eosinophils and neutrophils in BALF ↑ Serum IgE ↑ Lung IL-4, IL-5, IL-13, IL-17A, IFNγ↑ CD4 ^+^ IL-13^+^ Th2 cells and CD4 ^+^ IL-17A^+^ Th17 cells (82) ↑
BALB/c mice	HDM, 50 μg, i.t., day 0, 7	HDM, 50 μg, i.t., day 14	AHR ↑ Eosinophil, macrophage, lymphocyte, and neutrophil in BALF ↑ Serum total IgE and HDM-specific lgE ↑ IL-33, TNF-α, IL-6, IL-4, IL-5, IL-13, IL-17, IL-23, IL-31, eotaxin, CCL17 and CCL22 ↑ (83)
BALB/c mice	HDM (+aluminum hydroxide), 0.75 DU Der f and 0.75 DU Der p, i.p., days 0, 7 and 21	HDM, 0.75 DU Der f and 0.75 DU Der p, i.n., days 26, 27 and 28	Eosinophils in BALF ↑ Lung Tjp-1, Cldn-18↓, Lung Cldn-4, Gob-5, Muc5ac ↑ (66)
BALB/c mice	OVA (+aluminum hydroxide), 100 μg, i.p., day 1, 7, 14	OVA, 75 μg, LPS, 3.5 μg, i.t., day 21, 23, 25	IgE, peribronchial/perivascular lung inflammation, fibrosis, congestion, bronchial thickness ↑ Lung TNF-α, IL-1β, IL-5, IL-6, IL-4, IL-10, IL-13, MMP-13, iNOS, ICAM-1 ↑ (75)
BALB/c mice	OVA (+aluminum hydroxide), 10 μg, i.p., day 0, 7	1% aerosolized OVA, LPS, 0.005% w/v in the nebuliser, days 14, 15, 16	AHR ↑ Eosinophil, macrophage, and neutrophil in BALF ↑ Lung S100-A9, IL-12p40, MCP-1, RANTES ↑ (73)
C57BL/6 mice	OVA, 100 μg, i.t., LPS, 5 μg, i.n., day 1	OVA, 100 μg, i.n., LPS, 1 μg, i.n., day 7 and days 17–20.	Neutrophils (CD11b ^+^ Ly6G^+^), T cells (CD3 ^+^ MHCII^−^), B cells (B220 ^+^ MHCII^+^), dendritic cells (CD11c ^+^ SiglecF^−^ MHCII^+^), macrophages (CD11b ^+^ F4/80^+^) ↑ Lung IL-17A, TNF-α, IFN-γ, IL-6, IL-1α, CCL3/MIP-1α, CXCL1/CK, CCL20/MIP-1α, CCL-2/MCP-1, CCL-4/MIP-1β, CXCL-10, CXCL-9, CCL-5/RANTES, CXCL-13 ↑ (74)

## 3 IgE-mediated cell models

**Figure 2:**
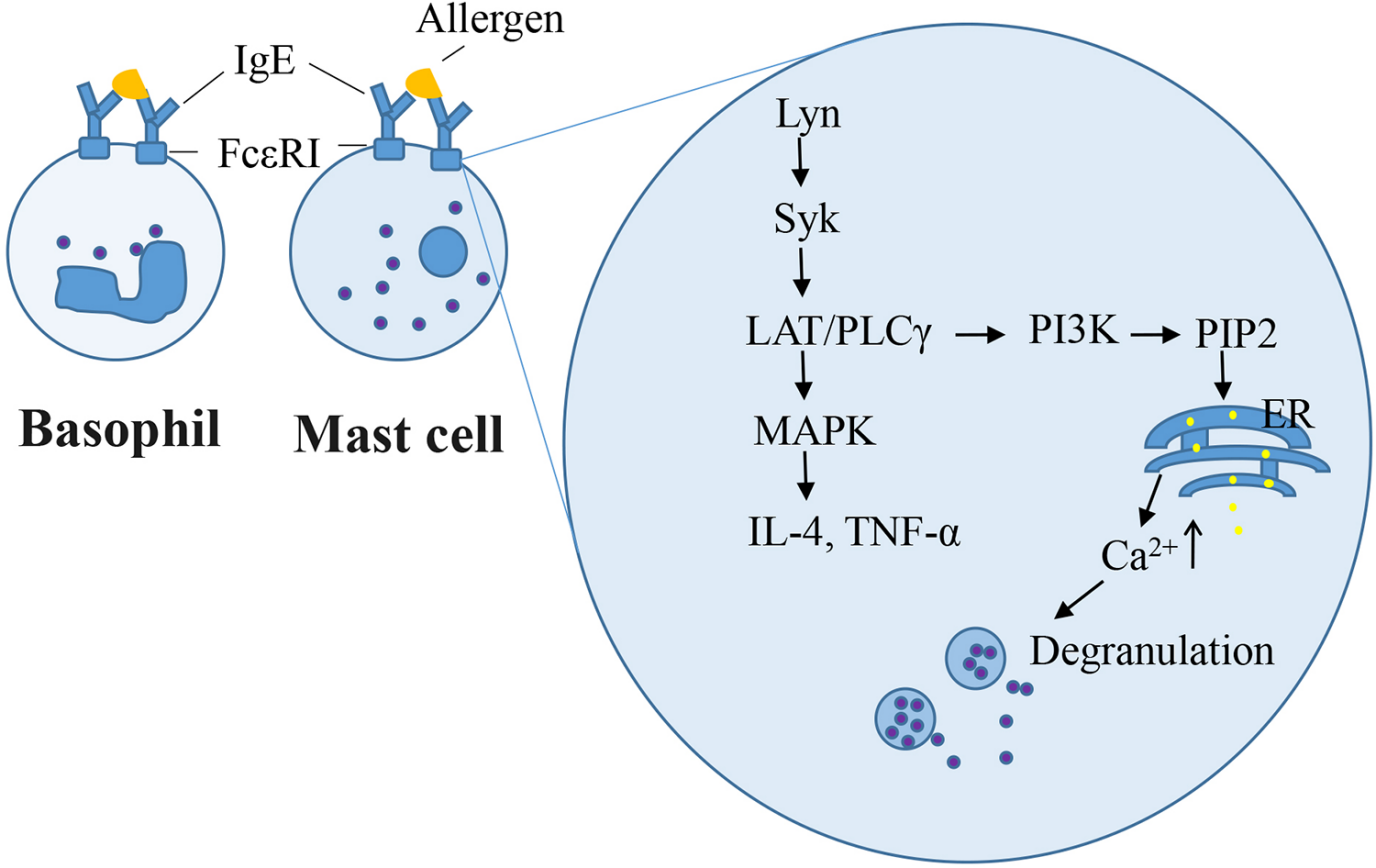
IgE-dependent mast cell degranulation.

The rat basophilic leukemia (RBL-2H3) cell line has been widely used as a mast cell model for *in vitro* studies because it mimics the mechanism of IgE-mediated degranulation of human mast cells. RBL-2H3 cell surface has abundant high-affinity IgE receptors (Fc*ε*RI), which bind with mouse/rat IgE (IgE-rich antiserum or anti-allergen IgE monoclonal antibodies) in *in vitro* experiments. Zhongcheng Liu constructed a stable hFc*ε*RI*α* (α-chain of human Fc*ε*RI)/RBL-2H3 cell line, which exhibits the species specificity in the interaction between IgE and Fc*ε*RI as that observed in humans (84). IgE-mediated RBL cell model is used to study of IgE–Fc*ε*RI interactions, intracellular signaling for degranulation and pro-inflammatory mediators, and novel anti-allergic drug screening. Degranulation and other mast cell responses were observed when cells were incubated overnight with anti-allergen IgE and challenged with allergens (59, 85, 86). Once IgE-antigen stimulation occurred on the cell surface, the level of phosphorylated Lyn, which is a Src family kinase, is enhanced, followed by the recruitment and activation of the tyrosine kinase Syk (87). This induces the LAT/PLC*γ* signaling pathway cascade and Ca^2+^ flux, which culminates in the exocytotic release of secretory granules (86). Other protein kinases, namely, Fyn and Fgr are required for the regulation of phospholipase D2 activation and degranulation (88). Furthermore, degranulation is a hallmark of immediate hypersensitivity reactions. The β-hexosaminidase activity is widely measured as a degranulation marker instead of histamine in IgE-sensitized RBL-2H3 cells. Late-phase Type I hypersensitivity reactions typically involve the formation of inflammatory mediators. Syk/LAT activation induces downstream MAPKs, which are involved in the production of IL-4 and TNF-α (89). In our previous study, RBL-2H3 cells were sensitized overnight using 2% anti-shrimp tropomyosin mouse serum and 100 ng/ml ST challenge for 1.5 h or 6 h, respectively, β-hexosaminidase release (33), or elevating the IL-4 level (59) in cells. Other cell lines, such as human mast cell line HMC-1, human peripheral blood basophilic leukemia cells KU812, and mouse mastocytoma P815 cell, lack Fc*ε*RI. The human leukemic mast cell line LAD2 possess Fc*ε*RI but still exhibits a very slow growth rate (90). Compound 48/80 or calcium ionophore induced-HMC1 or LAD2 cell degranulation model do not involve the initial events of Fc*ε*RI signaling upstream of Ca^2+^ influx. Primary cells such as rat peritoneal mast cells and mouse bone marrow-derived mast cells closely mimic *in vivo* biological responses. However, the quality of rat peritoneal mast cells remains insufficient. Mouse bone marrow-derived mast cells require 4–6 weeks for differentiation, maturation, and senescence after a brief culture period (91). IgE-dependent mast cell degranulation is shown in Figure 2.

## 4 Perspectives

Foreign allergens trigger Type I hypersensitivity, and few preventive therapies have been established apart from strict dietary/environmental exposure or allergen-specific immunotherapy. Thus, animal and cellular models are helpful for exploring potential molecular targets and screening drugs. An ideal animal model would require the same amount of allergen via the same route to induce sensitization and duration of exposure and present the same Th2 response as that in humans (92). Some biological agents that block Th2 response, such as anti-IL-4R*α* mAb, anti-IL-5/IL-5R mAb, anti-IgE mAb, are used for patients with allergic diseases (93). However, not all findings obtained from animal models may be replicated in human diseases. For example, although IL-17 blocking is effective in mouse models, a clinical trial using an anti-IL-17-receptor antibody failed to improve asthma exacerbation (94). Accordingly, the limitations of mouse models should be considered in the protocol design and interpretation of results.

In our previous studies, shrimp tropomyosin without alum adjuvant elicited a higher increase in total IgE levels in mouse sera compared with that of OVA, indicating greater allergenic potential. Various models, including shrimp tropomyosin-induced PCA, PSA, ASA, rhinitis/asthma mouse model, and RBL-2H3 cell degranulation model, have been established. Moreover, shrimp tropomyosin-induced asthma without adjuvants exhibited several hallmarks of human asthma, including high IgE production, AHR, eosinophilic inflammation, Th2 response, and airway remodeling. Owing to the advantage of sensitization without requiring an adjuvant, shrimp tropomyosin may be suitable for local sensitization when administered by intranasal or intratracheal instillation without an adjuvant, similar to that of HDM. In a future study, we plan to explore a modified protocol for establishing shrimp tropomyosin-induced asthma model and compare the allergenic potency between shrimp tropomyosin and HDM.
